# Characterization of Amino Acid Nanolayers and Their Interactions under Simulated Planetary Conditions

**DOI:** 10.1021/acsearthspacechem.4c00334

**Published:** 2025-02-11

**Authors:** Diogo Gonçalves, Florence Hofmann, Janina Drauschke, Severin Wipf, Riccardo Giovanni Urso, Ana M. Ferraria, Ana M. Botelho do Rego, Jana Bocková, Cornelia Meinert, Andreas Elsaesser, Bruno Pedras, Zita Martins

**Affiliations:** † Centro de Química Estrutural, Institute of Molecular Sciences and Department of Chemical Engineering, 72971 Instituto Superior Técnico, Universidade de Lisboa, Av. Rovisco Pais 1, 1049-001 Lisbon, Portugal; ‡ Institute for Bioengineering and Biosciences and Department of Chemical Engineering, 72971 Instituto Superior Técnico, Universidade de Lisboa, Av. Rovisco Pais 1, 1049-001 Lisbon, Portugal; § Freie Universität Berlin, Department of Physics, 9166 Experimental Biophysics and Space Science, Arnimallee 14, 14195 Berlin, Germany; ∥ 53294 INAF-Osservatorio Astrofisico di Catania, Via Santa Sofia 78, 95123 Catania, Italy; ⊥ Associate Laboratory i4HB-Institute for Health and Bioeconomy, 72971 Instituto Superior Técnico, Universidade de Lisboa, Av. Rovisco Pais 1, 1049-001 Lisbon, Portugal; # 27051 Université Côte d’Azur-CNRS, ICN, UMR7272, 06108 Nice, France

**Keywords:** alanine, glycine, planetary surfaces, Titan, mid-IR spectroscopy, AFM, nanoFTIR, XPS

## Abstract

Laboratory experiments extend our possibility to understand the behavior of organic molecules under extraterrestrial conditions. In the scope of such simulation experiments, organic molecules are often prepared as thin films, embedded in ice matrices, or adsorbed onto mineral surfaces. Albeit a single-species approach often adequately mimics the conditions to be studied, there are scenarios where the interactions between different organic molecules should be considered. In this work, we investigate the interaction of the two simplest α-amino acids, glycine and alanine, while codeposited as homogeneous nanolayers. Our results demonstrate that their interaction leads to deposition patterns, infrared signatures, and electronic properties that cannot be predicted by studying each molecular species in isolation. We conclude that organic interactions influence the photochemistry and spectroscopic signatures of biomolecules potentially present in planetary environments of interest such as Titan’s surface.

## Graphical abstract

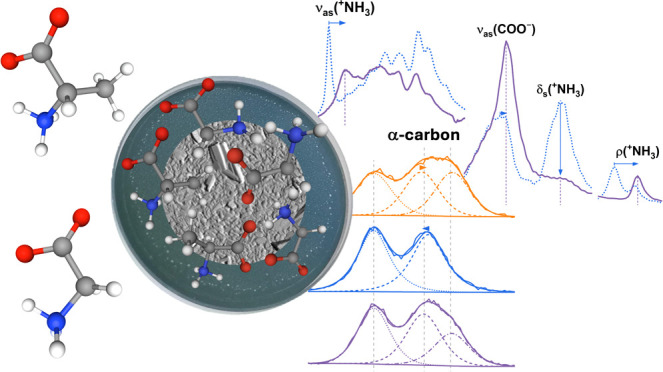

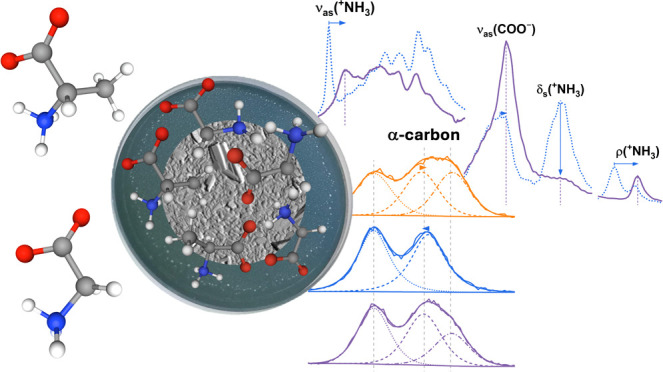

## 1 Introduction

The goal of understanding the origin and evolution of organic matter in different extraterrestrial environments has for long motivated astronomical observations, in situ and laboratory analyses of extraterrestrial samples (meteorites, asteroids, comets, and planets), and the use of simulation chambers in the laboratory. By surveying the conditions under which complex molecules are formed and maintained, we assess the role that exogenous delivery might have had on the origin of life on Earth,^1–5^ and constrain the distribution of the molecular building blocks of life elsewhere.^6–9^ Laboratory simulation experiments usually focus either on the formation of prebiotic molecules and their precursors^10,11^ or on their photostability in astrobiological environments of interest. There is a long-standing tradition of studying the photostability of organic compounds under Martian conditions,^12–18^ as recently reviewed by Wipf et al.,^19^ with the role of mineralogical composition frequently considered.^20–27^ The photochemical processes experienced by prebiotic molecules in space^28–35^ and icy conditions^36–43^ have also been the focus of extensive research.

In photostability studies, the organic molecule of interest is usually deposited on an inert, mid-infrared transparent substrate such as silicon^13,44^ or calcium fluoride (CaF_2_) windows,^45^ diluted in a potassium bromide (KBr) pellet, adsorbed onto a mineral surface, or embedded in an ice matrix. Albeit some researchers have opted to simultaneously irradiate different amino acids adsorbed to the same mineral substrate^26,46,47^ or embedded in the same ice matrix,^36^ the influence of their intermolecular interactions on their photochemistry has never been, to the extent of our knowledge, the target of study. Despite that, some results have already suggested that organic interactions may yield photoprotective effects.^48^

A deeper understanding of how surrounding organic interactions influence individual molecules would be highly valuable in advancing research in both astrobiology and astrochemistry. It could, for instance, enrich scenarios on extraterrestrial organic preservation,^49,50^ as the knowledge on organic-mineral interactions has done.^20,24–26^ Further, it would complement the search for organics on extraterrestrial surfaces, in which the influence of inorganic matrices was mainly considered.^20,48,51^ Moreover, it could help to constrain the distribution of prebiotic molecules in the interstellar medium (ISM) and the composition of the organic refractory material in which it is found.^52–56^ Besides, on Titan’s surface, hydrolysis of the superficial organic layer by water–ammonia cryomagma flows could form prebiotic molecules.^57–60^ However, due to the limited diffusion of these molecules into the thick, slurry-like cryomagma,^61,62^ they are likely confined to the interface with the water–ammonia flow, potentially persisting as organic films. To assist NASA’s Dragonfly mission in investigating Titan’s cryovolcanic features,^63^ it is essential to constrain the chemical evolution and spectroscopic signatures of these complex organic mixtures.

In this work, we discuss how the interaction between the two simplest α-amino acids may influence their spectroscopic signatures and physicochemical properties. Through vacuum sublimation we have deposited nanolayers of homogeneous mixtures of racemic alanine and glycine (alanine + glycine) at a 1:1 molar ratio. At this mixing proportion, key glycine and alanine vibrational modes present similar infrared (IR) absorbances, with which we have characterized the behavior of the individual molecules at room temperature and upon cooling. We have also characterized the morphology of the mixture layer through atomic force microscopy (AFM), its spatial features through nanoscale Fourier-transform infrared spectroscopy (nanoFTIR), and its electronic properties and atomic composition (except for hydrogen) through X-ray photoelectron spectroscopy (XPS). At each step, we compared our results with those from similarly produced nanolayers of the individual amino acids. The interactions discussed in this work may affect other secondary processes such as phase transformations,^43,44,64^ desorption processes,^65^ and the products of thermal and aqueous alteration.^66,67^ In a separate work we show that the interaction between alanine and glycine sharply affects their ultraviolet photochemistry.^68^ In particular, we demonstrate that glycine photodegrades ten times faster in a alanine + glycine mixture nanolayer (herein characterized) than in a pure glycine nanolayer. This unexpected behavior motivated the present characterization of the alanine-glycine interaction. The extensive study of the ready formation in space conditions^69–71^ and presence in carbonaceous chondrites^29,72^ of alanine and glycine has provided us solid ground on which to evaluate the consequences of their interaction.^20,43,44,73–75^

## 2 Materials and Methods

### 2.1 Sample Preparation

Glycine (Sigma-Aldrich, 99.7% purity), *rac*-alanine (TCI, 98.5% purity), and alanine + glycine nanolayers were deposited by vacuum sublimation onto calcium fluoride (CaF_2_) windows (20 mm diameter, 2 mm thick, Korth Kristalle GmbH) from resistively heated polytetrafluoroethylene (PTFE) crucibles at 10^–6^–10^–5^ mbar. 150 mg of the deposited organic material was added to the crucible. Prior to deposition, the material was prepurified by heating it for 15 min at 80 °C under vacuum, with the windows masked by a mobile shutter. All depositions were made at constant temperatures: 110 °C (glycine) and 115 °C (alanine and alanine + glycine), ensuring slow sublimation (1–2 Å s^–1^) and homogeneous deposition onto the windows placed above the crucible. Film thicknesses were monitored using a quartz crystal microbalance, targeting thicknesses no higher than 100 nm.

In the mixture nanolayer, the alanine-to-glycine ratio was tuned by the mass of each amino acid added to the crucible, while accounting for their respective deposition rates at the selected temperature. The desired 1:1 molar ratio, as measured by XPS, was produced by the addition of thoroughly mixed 122 mg of *rac*-alanine and 28 mg of glycine to the crucible. To minimize amino acid contamination, the interior surfaces of the vacuum chamber were coated with aluminum foil, regularly renewed, and the CaF_2_ windows were solvent- and plasma-cleaned before usage.

### 2.2 IR-Transmission Spectroscopy

The absorption spectrum of all samples was measured in transmission mode at room temperature inside a controlled nitrogen environment (Glovebox, MBraun), after 1 min exposure to ambient atmosphere, using an ArcOptix OEM FT-IR spectrometer (5000–832 cm^–1^) at 4 cm^–1^ resolution averaged over 20 scans. This spectrometer features an internal light source (SiC globar) and an MCT (4-TE cooled) detector.

The low-temperature studies were performed in an ultrahigh vacuum (UHV) chamber (*P* ≤ 10^–8^ mbar) hosting a sample holder in thermal contact with the coldfinger of a closed-cycle helium cryocooler (ARS). The samples were fitted to the sample holder at room pressure and temperature and then cooled down to 90 at 3 K min^–1^ in UHV conditions. Transmission spectra were collected throughout the cooling process with an ArcOptix OEM FT-IR spectrometer. The IR beam passes through the zinc selenide (ZnSe) windows of the UHV chamber and arrives at normal incidence with respect to the sample surface. Both the UHV chamber and the IR spectrometer are hosted in a nitrogen-purged environment to reduce the atmospheric contributions. Spectra were recorded throughout the cooldown in the range 5000–834 cm^–1^ with a resolution of 0.5 cm^–1^, coadding 30 scans for each spectrum. The absorption spectra at different temperatures were calculated using empty CaF_2_ windows under the same conditions. The spectra were smoothed using a Savitzky-Golay method with window size 40 and polynomial order 2.

### 2.3 Atomic Force Microscopy (AFM) and Nanoscale Fourier-Transform Infrared Spectroscopy (nanoFTIR)

AFM images were acquired using a neaSNOM Nearfield Microscope (Neaspec, Germany) with coated AFM probe tips (Arrow NCPt, NanoWorld, Switzerland) possessing a resonance frequency at ≃285 kHz. The metrological numerical analyses were averaged over two 5 × 20 μm^2^ or 5 × 18 μm^2^ scan areas for each layer. Those scan areas included exposed CaF_2_ substrate, whose organic layer was removed with a sharp plastic tip, meant to measure the organic layer thickness by its average height difference to the CaF_2_ substrate. The root-mean-square (RMS) roughness was computed through the standard deviation of the layer heights. Its margin of error was the RMS of the bare substrate. The same margin of error was used for the maximum layer height above the average substrate level. The margin of error of the average layer height (relative to the average substrate) was given by the sum of the two roughness values (layer and substrate). Visualization and analysis were performed with Gwyddion (v.2.65, Czech Metrology Institute, Czech Republic).

NanoFTIR spectra were acquired in six locations of each sample using the spectroscopy module of the neaSNOM Nearfield Microscope (Neaspec, Germany) and a 4.5–15 μm (≃2222–667 cm^–1^) broadband laser source. Three of the probed features located above 70% of the height range within the 5 × 5 μm^2^ scan, and three others below 40% of the same range. At each position, five spectra were acquired, each derived from 10 scans with a 6 ms integration time and 9 cm^–1^ resolution. NanoFTIR absorption spectra were calculated from the second harmonic phase and amplitude signals.^76,77^ Their margin error was assumed to be the standard variation within the five nanoFTIR absorption spectra for each location.

### 2.4 IR-Reflectance Microscopy

To assist the analysis of the band shapes and peak positions in the nanoFTIR spectra, IR-reflectance microscopy was performed for all samples, using an IR-reflectance microscope (Hyperion 2000, IR source provided by a Vertex 80v, Bruker, Germany) fitted with a 15× Cassegrain objective (NA = 0.4, Newport, Germany). The amino acid nanolayers were deposited on gold substrates using the vacuum sublimation protocol described above. The reflection–absorption spectra were collected at normal incidence and background-corrected against the spectrum of the gold substrate, averaged over 500 scans. Each sample spectrum was acquired from an average of 25 points, equally spaced by 10 μm in a 5 × 5 matrix, performing 500 scans at each point, from 3950 to 650 cm^–1^ at 4 cm^–1^ resolution. The margin of error of each sample spectrum was assumed to be the standard variation within the 25 points.

### 2.5 X-ray Photoelectron Spectroscopy (XPS)

Samples were chemically characterized by XPS using a XSAM800 dual anode spectrometer from Kratos. The Mg Kα radiation (*h*ν = 1253.6 eV) was selected. Spectrometer operation conditions, TOA, pressure and temperature of analysis were as described by do Rego et al.^78^ Spectra (with a step of 0.1 eV) were collected using the software Vision 2 for Windows, Version 2.2.9 from KRATOS. Data processing was performed using the freeware XPSPeak 4.1. Shirley backgrounds and Gaussian–Lorentzian products (GL%) were used for curve fitting. No flood gun was used for neutralizing charge accumulation. For calibration purposes we have used a nanolayer sample of a racemic mixture of α-aminobutyric acid from d-α-aminobutyric acid (Acros Organic, 99% purity) and l-α-aminobutyric acid (Acros Organic, 98% purity), produced as the alanine and glycine samples described above with a deposition temperature of 102 °C. The shift due to charge accumulation was corrected using as reference the lower binding energy (BE) found for the C 1s region of the α-aminobutyric acid set to 285.0 eV and assigned to the carbon in the −CH_3_ group.^79^ Using this reference (spectra not shown), the carboxylate group was found at a BE = 288.06 eV. This value was used as reference for the alanine, glycine, and alanine + glycine samples. For quantification purposes, the sensitivity factors (SF) used were those provided by the equipment library: 0.318 for C 1s, 0.736 for O 1s, 0.505 for N 1s, 1 for F 1s and 1.95 for Ca 2p.

## 3 Results and Discussion

### 3.1 IR Characterization

IR spectroscopy is commonly applied to characterize amino acids produced from ice photochemistry and to identify them in space. In this work, we used transmission FT-IR spectra to shed light on the extent of the interaction between the two amino acids and the spectral tendencies it may produce. In fact, vibrational mode bands change significantly when varying the chemical environment of each molecule.^43,44,80^ In this section, we explore the spectral changes that alanine and glycine undergo when codeposited.

#### 3.1.1 Alanine and Glycine Samples

The transmission FT-IR measurements of the alanine and glycine samples agree with previous measurements on solid phase samples.^43,44^ Having deposited the nanolayers on the CaF_2_ substrate at room temperature, we obtained, as expected, both molecules as zwitterions (with positively charged ammonium, –^+^NH_3_, and negatively charged carboxylate, −COO^–^, groups), with no evidence of any fraction of molecules in the neutral state (Table 1). The same observation, confirmed by XPS in Section 3.3, applies to the alanine + glycine sample.

**Table 1:** Assignment of the Distinguishable Bands of the Infrared Spectra of Alanine, Glycine and Alanine + Glycine Samples at Room Temperature

	**alanine**		**glycine**		
assignment^a^	this work^b^ ν̅ (cm^–1^)	dl-alanine^c^ ν̅ (cm^–1^)	this work^b^ ν̅ (cm^–1^)	β-glycine^a^ ν̅ (cm^–1^)	**alanine + glycine ν̅ (cm^–1^)**
ν(CC)			894^a,b^	894	
ρ(CH_2_)			916^a,b^	916	
τ(^+^NH_3_)	926^c^	919			
ρ(^+^NH_3_)	1012^c^	1015, 1027			1014
ν_as_(CCN)			1040^a,b^	1041	1042
ρ(^+^NH_3_)	1110^c^	1114	1118^a^, 1136^a,b^	1120, 1135	1116
δ(CH)	1134^c^	1149, 1165			
ρ(CH)	1230^c^	1238			1238
δ(CH)	1306^c^	1307			1308
τ(CH_2_)			1310^a^		
ω(CH_2_)			1336^a,b^	1336	1330
δ_s_(CH_3_)	1356^c^, 1366^c^	1354			1360
ν_s_(COO^–^)	1412^c^	1409	1414^a,b,d^	1416	1412
δ(CH_2_)			1446^a,d^	1445	1442
δ_as_(CH_3_)	1454^c^	1453			1450
δ_s_(^+^NH_3_)	1522^c^	1530	1522^a,d^	1526, 1543, 1562	1522
ν_as_(COO^–^)	1588^c^	1588	1602^a,d^	1606	1594
δ_as_(^+^NH_3_)	1634^c^	1621	1664^a,d^	1655	
δ_as_(^+^NH_3_) + τ(^+^NH_3_)	2108^c,e^	2124	2134^a,e^	2136	2116
ν_s_(^+^NH_3_), ν_s_(C–H)	2530–3000^c^	2920, 2986, 3002	2530–3000^a^	2969, 3006	2530–3000
ν_as_(^+^NH_3_)	3044^c^	3077	3180^d^	3188	3076

a ν, bond stretching; ρ, rocking; τ, torsion; δ, scissoring; ω, wagging; as, antisymmetric; s, symmetric.

b Assignment based on: a. Chernobai et al.^74^; b. Maté et al.^43^; c. Minkov et al.^73^; d. Liu et al.^75^; e. Jarmelo et al.^81^

The deposition by vacuum sublimation of glycine should produce the metastable β polymorph over its alternative α and γ forms.^75^ Indeed, comparing the absorption spectrum of the deposited glycine layer with the spectra of the three pure glycine polymorphs,^74^ we find a good match with the vibrational energies reported for β-glycine. We obtained an equally good match between the alanine vibrational energies and those previously reported for racemic alanine.^73^

The alanine sample presents broader IR absorption bands than glycine (Figure 1), which is compatible with the amorphicity of the alanine nanolayer detected in the AFM studies, detailed in Section 3.2. When deposited to a 700 nm thickness, however, the alanine absorption spectrum attains sharp features as those measured in the glycine spectrum, without any shift in vibrational energies. This hints that *rac*-alanine does not crystallize as readily as glycine at such thin nanolayer scales, requiring higher sample thickness for more significant crystallization. The excellent fit between the absorption spectrum of the alanine nanolayer and the normalized 700 nm spectrum (Figure S1) further confirms that, despite its amorphous structure, the alanine sample has the expected amount of material for a 100 nm layer.

**Figure 1:**
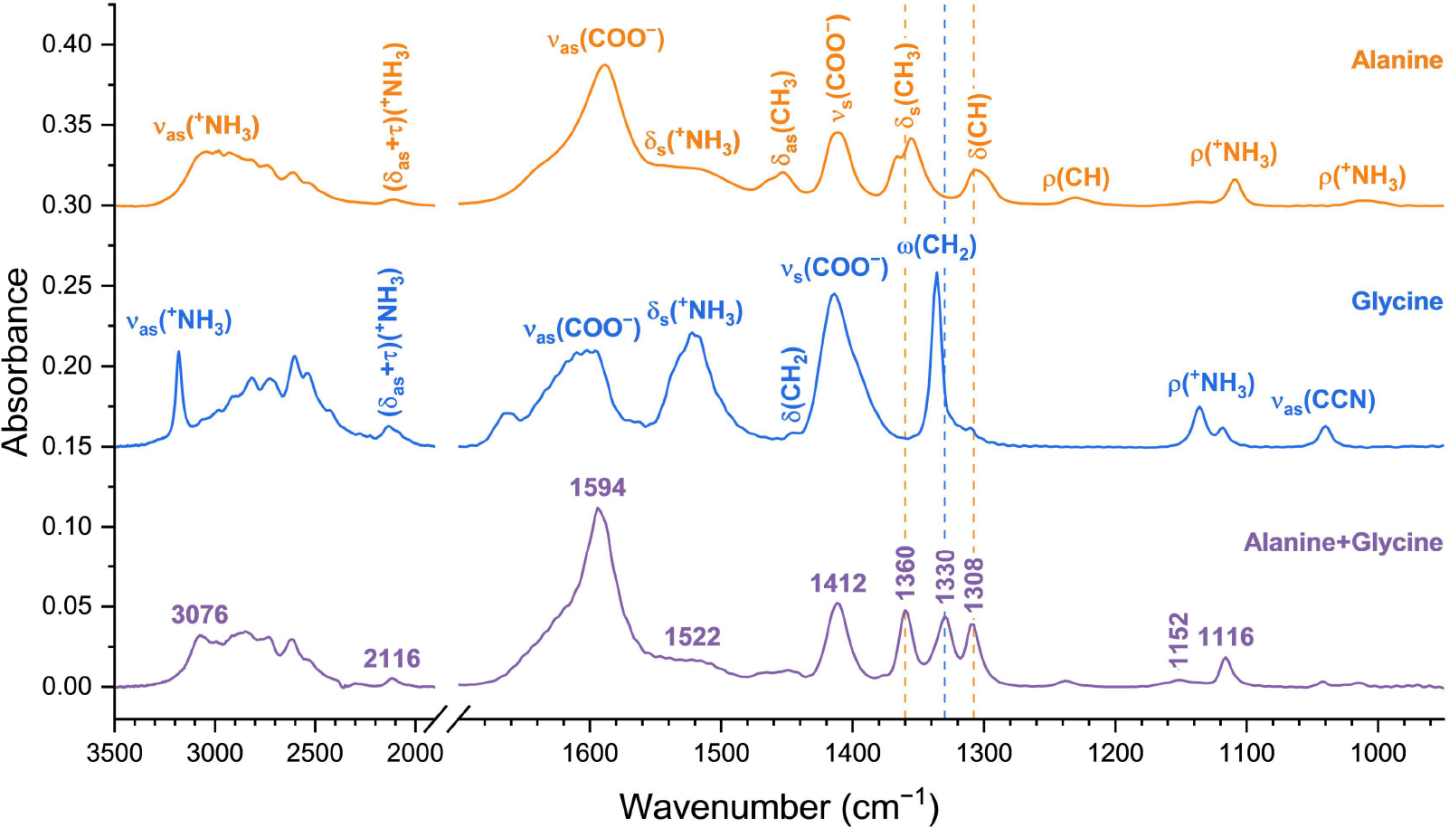
Transmission FT-IR spectra of the pure alanine, pure glycine and alanine + glycine mixture (1:1 molar ratio) nanolayers. Despite slight shifts in energy and changes in band shapes, the spectrum of the alanine + glycine mixture displays the ω­(CH_2_) mode, specific to glycine, and the δ_s_(CH_3_) and δ­(CH) modes, specific to alanine, that allow to distinguish the presence of both molecules upon codeposition.

The region from 1275 to 1375 cm^–1^ best differentiates the alanine from the glycine sample. Whereas the glycine spectrum shows a single absorption band at 1336 cm^–1^, assigned to the wagging (ω) mode of its −CH_2_– group, the alanine sample presents two bands at 1306 and 1356/1366 cm^–1^, encoding for scissoring (δ) modes of its −CH– and −CH_3_ groups. Crucially, in the transmission FT-IR spectra of the nanolayers, these three molecule-specific peaks are sufficiently separated to allow easy assignment when codeposited in the mixture sample (Figure 1).

#### 3.1.2 Alanine + Glycine Sample

The 1:1 mixing ratio resulted in comparable absorbances for key infrared bands of glycine and alanine, namely for those molecule-specific bands—assigned to the −CH_2_– group of glycine and to the −CH– and −CH_3_ groups of alanine (Figure 1). The comparable absorbances allowed us to assign the alanine + glycine infrared bands by drawing an analogy to the vibrational modes measured in the individual amino acids. By proximity in energy and/or band shape we could confidently assign all but one of the transitions observed in the alanine + glycine spectrum. An unassigned band found at 1152 cm^–1^ likely encodes for a new composite vibration resulting from the bimolecular arrangement.

##### 3.1.2.1 Alanine- and Glycine-Specific Groups

Looking at the changes in the vibrational structure of alanine, the environment of its −CH_3_ and −CH– groups alters significantly from the alanine to alanine + glycine mixture sample. The alanine + glycine absorption band centered at 1360 cm^–1^ has no equivalent in the glycine spectrum, and we can thus safely assign it to the δ_s_(CH_3_) vibrational mode that in the alanine sample is asymmetrically split into two peaks at 1356 and 1366 cm^–1^ (averaged at 1361 cm^–1^). Although the band maximum in the alanine + glycine sample (1360 cm^–1^) compares well with the peak-average in the alanine sample (1361 cm^–1^), the absence of any splitting in the former hints to the −CH_3_ alanine group becoming spatially flexible when codeposited with glycine molecules, which is compatible with an increased spatial freedom suggested by the slight broadening of the δ_as_(CH_3_) mode. Regarding the alanine −CH– group, its ρ­(CH) vibrational mode blue-shifts from 1230 to 1238 cm^–1^, as does the δ­(CH) mode from 1306 to 1308 cm^–1^. The most significant change, however, lies on how the latter sharpens from its broad, asymmetrical shape to a well-defined symmetrical band. This transformation is likely due to the higher degree of crystallinity of the alanine + glycine sample relative to the alanine sample (as discussed in Section 3.2). The evolution seen in the −CH_3_ group modes—likely caused by a lower physical hindrance—and in the −CH– group—likely due to a higher spatial hindrance of the α-carbon—demonstrates how the skeleton of alanine is differently packed in the presence of glycine.

The glycine-specific groups experience a reciprocal, although more limited, effect when combined with alanine. Albeit the ν_as_(CCN) mode remains unchanged (with a negligible blueshift from 1040 cm^–1^ in the glycine to 1042 cm^–1^ in the alanine + glycine sample), the ω­(CH_2_) mode red-shifts from 1336 to 1330 cm^–1^, retaining its sharp spectral shape. We have assigned the scissoring (δ) mode of the −CH_2_– group to the shoulder band at 1442 cm^–1^, which did not shift considerably from 1444 cm^–1^ in the glycine sample. The limited effects on its skeleton upon codeposition with alanine hint to similar packing of glycine compared with its individual deposition. These observations suggest that upon codeposition of the two amino acids, glycine induces more crystallinity in deposited alanine than having the latter inducing its amorphicity. This evolution is reproduced when we compare the alanine + glycine spectrum to the spectrum-average of the pure alanine and pure glycine spectra (the spectrum expected in the absence of alanine-glycine interactions), Figure S2. This comparison (Figure S2) clearly illustrates the spectroscopic signatures produced by the copresence of both amino acids and is further described in SI.

##### 3.1.2.2 Ionized Groups (−^+^NH3 and −COO^–^)

The crystallizing influence that glycine induces in alanine also affects the ρ­(^+^NH_3_) vibrational mode of alanine, measured at 1014 cm^–1^ in the alanine + glycine sample and assigned to the 1012 cm^–1^ band in the alanine sample. In this spectral region, this band has no equivalent in the glycine sample. The codeposition with glycine sharpens this absorption band, with its full width at half-maximum (fwhm) reducing by 59.1% from 27.9 to 11.4 cm^–1^.

Albeit the ionized groups (−^+^NH_3_ and −COO^–^) are common to both amino acids, their vibrational modes occur at slightly different energies in the spectra of the single-species samples. Interestingly, when the amino acids are mixed, these common modes combine into single absorption bands, with the vibrational energy of the combination band falling in between the vibrational energies measured in the alanine and glycine samples. No band at the previous energies (those in the pure alanine and pure glycine spectra) are found in the alanine + glycine spectrum. This behavior is exemplified by the absorption band detected at 1116 cm^–1^ in the alanine + glycine spectrum. Both the alanine and glycine samples show bands corresponding to the rocking (ρ) vibration of the –^+^NH_3_ group, albeit at slightly different energies: 1110 cm^–1^ in the alanine sample, and as an asymmetrical doublet with maxima at 1118 and 1136 cm^–1^ (averaged at 1127 cm^–1^) in the glycine sample. The band at 1116 cm^–1^ in the alanine + glycine sample, located between the alanine band (at 1110 cm^–1^) and the averaged glycine band (1127 cm^–1^), with no other sharp bands in its vicinity, supports our assignment of this band to the combined rocking vibration of the –^+^NH_3_ groups from both molecules (Figure 2). Additionally, the band at 2116 cm^–1^ in the alanine + glycine sample lies between the corresponding glycine (maximum at 2134 cm^–1^) and alanine (maximum at 2108 cm^–1^) bands, all representing a combination of antisymmetric scissoring and torsional modes of the –^+^NH_3_ group.^81^ Further, the very broad absorption envelope from 2300 to 3300 cm^–1^ encompasses the C–H and the N–H stretching bands of glycine and alanine. The contribution of both amino acids to this region is evident from the shape of the absorption envelope, which combines spectral features from each amino acid (Figure 2). Here, we observe the highest-energy band at 3076 cm^–1^ in the alanine + glycine sample. This band has a sharper equivalent in the glycine sample (at 3180 cm^–1^) and a broader one in the alanine sample (at 3044 cm^–1^), leading us to assign this band in the alanine + glycine spectrum to the ν_as_(^+^NH_3_) vibrational mode shared by both molecules. The fact that the band position is not predicted by the normalized sum of the pure alanine and pure glycine spectra (Figure S2) conclusively demonstrates the combination of the vibrational modes from glycine and alanine. Since this vibrational mode serves as a proxy for hydrogen bond strength,^73^ we conclude that the average hydrogen bond strength in the mixture falls between that of alanine and glycine, though it is closer to the former. This vibrational mode exemplifies the trend observed in all ^+^NH_3_ modes above: upon mixing, the alanine and glycine –^+^NH_3_ groups do not exist independently but vibrate in combined vibrational modes, with energies closer to those of the pure alanine sample.

**Figure 2:**
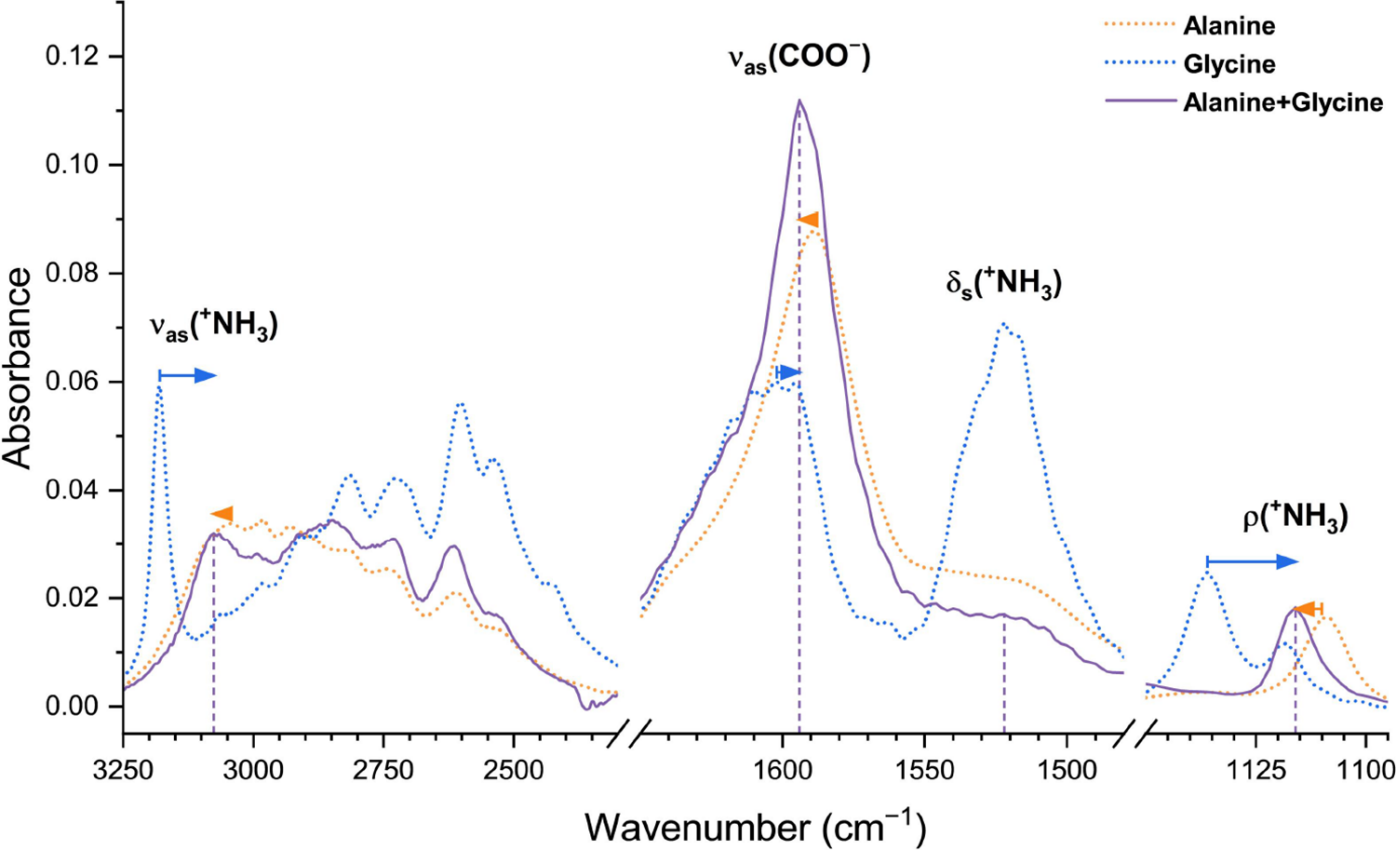
Evolution of the vibrational modes of the functional groups common to glycine and alanine (−^+^NH_3_ and −COO^–^) upon codeposition. Such vibrational modes combine into one, intermediate in energy and band shape between the individual amino acids, despite being closer to alanine in both parameters. The dominance by the alanine properties in the mixture is clearest in the antisymmetric stretching (ν_as_) (3076 cm^–1^) and rocking (ρ) (1116 cm^–1^) modes of the –^+^NH_3_ group, at which the vibrational energies of pure alanine and pure glycine differed the most.

In the combined modes, the band shape also remains slightly closer to that of alanine. This is clear in the antisymmetric stretching of the −COO^–^ group: this mode presents a broad shape in the glycine sample (maximum at 1602 cm^–1^) but a sharp shape in the alanine (centered at 1588 cm^–1^) and, crucially, in the alanine + glycine sample (centered at 1594 cm^–1^)—Figure 2. The dominance of the alanine shape over the glycine shape is replicated in three other instances: the singlet band assigned to the ρ­(^+^NH_3_) mode at 1116 cm^–1^, the shoulder band assigned to the δ_s_(^+^NH_3_) at 1522 cm^–1^ and the highest-energy broad ν_as_(^+^NH_3_) band at 3076 cm^–1^ (Figure 2).

We attribute this band coalescence to an intermolecular vibrational coupling^82^ of the vibrational modes shared by alanine and glycine. Vibrational couplings are known to influence the spectra of molecular crystals,^83–85^ namely those of pure glycine.^86^ In our case, the coupling of the ^+^NH_3_ and COO^–^ vibrational modes suggest an ordered interaction between the two amino acids in crystal domains^85,87^ within the alanine + glycine nanolayer, in an arrangement that neither alanine nor glycine could achieve independently. The fact that both molecules adopt a different configuration upon mixing is further supported by the density of the alanine + glycine layer, as it is not the weighted average of those of the pure alanine and pure glycine nanolayers, but, instead, similar to the latter, as detailed in Section 3.3.

Overall, whereas glycine enforces alanine to a more rigid arrangement of the molecular skeletons, alanine largely dictates the strength of the vibrational modes of the ionized groups and, ultimately, the strength of the hydrogen bonds between the –^+^NH_3_ and −COO^–^ groups. As, when deposited separately, alanine molecules form stronger hydrogen bonds compared to glycine molecules,^73^ upon codeposition, alanine causes glycine to experience stronger intermolecular hydrogen bonding than it would when deposited alone.

In astrobiological environments of interest, amino acids accumulate and interact at temperatures far below room temperature. On Mars, temperatures can be as low as 150 K,^88^ while the organic-rich surface of Titan shows an average temperature of 94 K.^89^ To understand whether the room temperature observations mentioned above hold in cooler planetary surfaces, we have also analyzed the transmission FT-IR spectra at temperatures down to 90 K. As described in SI, upon cooling, the bands in the alanine + glycine spectrum evolved similarly to those in the individual amino acid samples, lacking any anomalous behavior. Crucially, the band sharpening produced during cooling (Figure S3) did not uncover separate contributions from alanine and glycine to the combined bands (Figure 2). This confirms that the latter are not thermal broadening artifacts but indeed illustrate the vibrational coupling of the ionized groups of alanine and glycine.

### 3.2 Topographic Characterization

To grasp the consequences of the new bimolecular arrangement, the topography of the three samples (alanine, glycine, and alanine + glycine) was analyzed through AFM. Starting with the single-species samples, we found a clear difference between the arrangement of the pure alanine and pure glycine nanolayers. While the alanine sample was arranged in irregularly sized grains, the glycine sample presented a lamellar crystalline arrangement of its molecules (Figure 3a). Area analyses of the single-amino acid nanolayers revealed a highly irregular alanine surface with an RMS roughness of 29.8 ± 8.8 nm, contrasting with flat glycine plateaus with about half the roughness of the former, 16.6 ± 6.6 nm.

**Figure 3:**
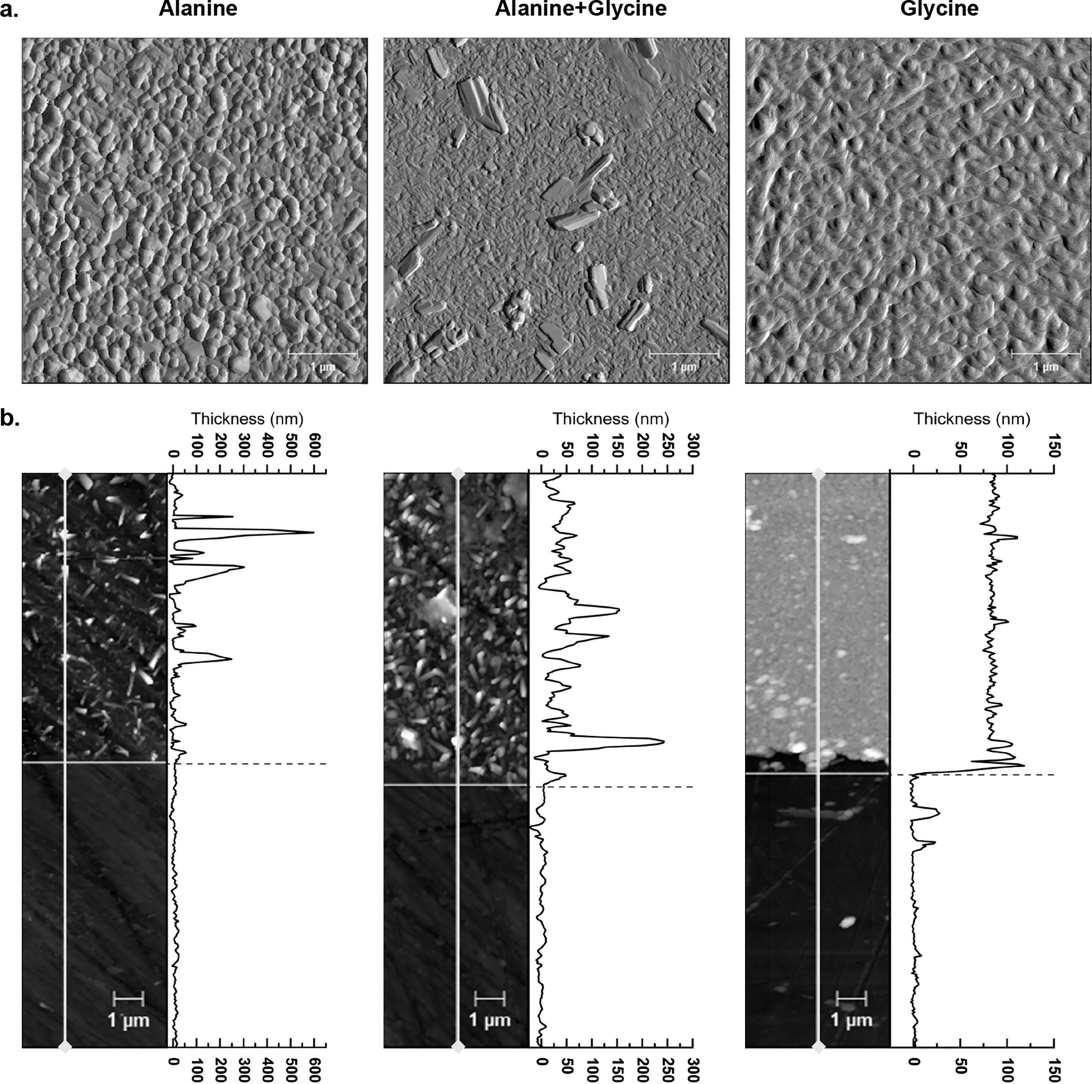
AFM surface images of pure alanine (left), pure glycine (right) and the alanine + glycine mixture (center) nanolayers. (a) Surface 5 × 5 μm^2^ scan windows show how the structure of the nanolayers progress from amorphous (pure alanine) to lamellar crystalline (pure glycine) arrangements, with the alanine + glycine arrangement falling in-between the two. (b) Surface topography of alanine (5 × 20 μm^2^), glycine (5 × 20 μm^2^) and alanine + glycine (5 × 18 μm^2^) with exposed CaF_2_ substrate. A height profile along the longest dimension (gray lines) is depicted on the right of each surface image. The glycine nanolayer presents low roughness and a well-defined step transition to the CaF_2_ substrate, in contrast with the alanine nanolayer. The properties of the alanine + glycine nanolayer, particularly its maximum peak height, fall in-between those of the single-species samples.

While the glycine layers exhibited a well-defined thickness, the alanine agglomerated arrangement lacked a clear step transition between the substrate and the organic layer (Figure 3b). By measuring the difference between the average heights of the layer and the CaF_2_ substrate, the glycine layer returned an average thickness of 80.7 ± 23.1 nm, and the alanine layer an average thickness of 8.1 ± 38.6 nm. The difference between the thicknesses of the alanine and the glycine nanolayers did not agree with the transmission FT-IR measurements. Specifically, the absorbance of the carboxylate group (−COO^–^) stretching mode, commonly used to quantify amino acids,^39,41^ indicated that the amount of alanine was only 18% lower than that of glycine. This contrasts with the 90% difference inferred from the measured thicknesses. Given this discrepancy, as well as the uncertainty in the thickness measurements due to the significant surface layer roughness (Figure 3b), we hold little confidence in the accuracy of the thickness measurement for alanine.

Upon codeposition of the two amino acids, the alanine + glycine mixture layer presented intermediate topographic properties between the alanine and the glycine samples. A 5 × 5 μm^2^ surface image of the alanine + glycine sample revealed a surface sprinkled with large lamellar crystals (Figure 3a). By characterizing the superficial features of the alanine + glycine sample through nanoFTIR, we understood that the lamellar crystals, uniformly distributed throughout the sample surface, were enriched in glycine. Still, the glycine molecules were not segregated from alanine, as the bulk composition of the alanine + glycine sample (i.e., those locations which did not include superficial crystals) mixed alanine and glycine at the same ratios as those inferred through transmission FT-IR (Figure S5). The nanoFTIR analysis was backed by IR-reflectance microscopy and is discussed at length in SI.

The alanine + glycine sample presented a RMS roughness higher than the one measured for pure alanine (40.7 ± 7.7 vs 29.8 ± 8.8 nm), but lower maximum heights above the average substrate height (255.4 ± 7.7 nm vs 440.3 ± 8.8 nm). The alanine + glycine layer still lacked the distinct step transition between the substrate and the organic layer seen in the glycine sample, but, with the transition being clearer than in the alanine layer, it returned a more representative average thickness of 26.3 ± 48.4 nm (Figure 3b). Overall, the codeposition of the two amino acids produced layers whose topographic properties fall in-between the two unexpected opposites set by the pure alanine and pure glycine samples, in a reciprocal influence compatible with that portrayed by their IR spectra.

### 3.3 XPS Characterization

To understand whether the interactions described in the previous sections impact the intramolecular electronic properties of alanine and glycine, we analyzed the three samples (alanine, glycine, and alanine + glycine) by X-ray photoelectron spectroscopy (XPS). Wide spectra (not shown) show that all samples contain mainly carbon, nitrogen and oxygen as expected. Fluorine and calcium contributions due to the CaF_2_ substrates were detected in trace amounts. Figure 4 displays the detailed C 1s, O 1s and N 1s regions for the alanine, glycine and alanine + glycine samples. Table 2 summarizes the binding energies and atomic percentages for all the peaks fitted to each region.

**Figure 4:**
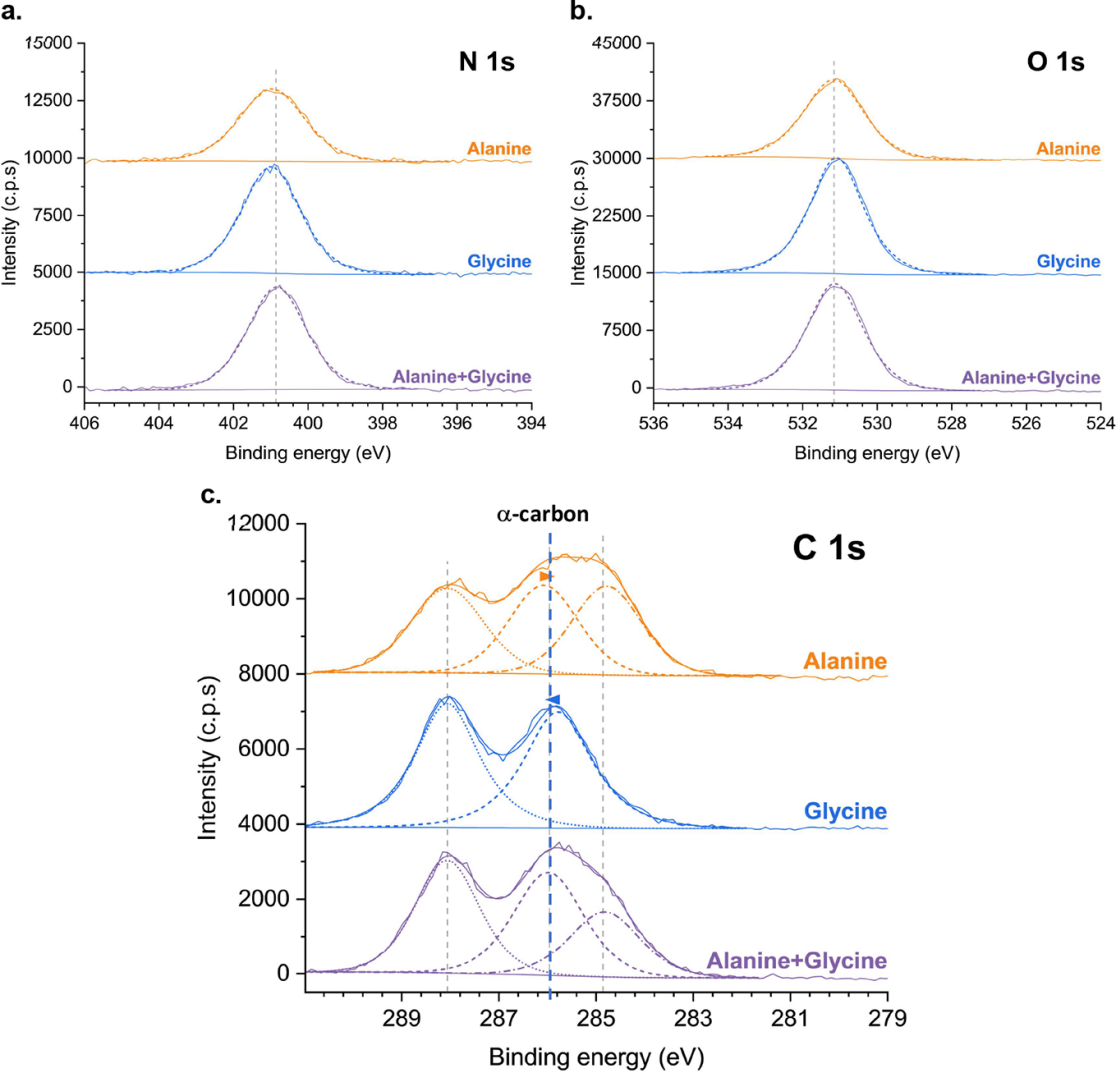
Detailed XPS N 1s (a), O 1s (b) and C 1s (c) regions for the alanine, glycine and alanine + glycine nanolayers. Spectra were offset for clarity of presentation. (c) The α-carbons of alanine and glycine become indistinguishable in the alanine + glycine nanolayer after their individual BE converge.

**Table 2:** Binding Energy (BE), in eV (± 0.1 eV), and Atomic Percentages (atom %) (± 5%) Resulting from the Peak Fitting Presented in Figure 4 ^a^

	alanine		glycine		alanine + glycine	
	BE (eV)	atom (%)	BE (eV)	atom (%)	BE (eV)	atom (%)
C 1s 1^b^						
C 1s 2	284.8	16.3			284.6	8.6
C 1s 3	286.1	16.3	285.8	19.7	285.9	17.7
C 1s 4	288.1	16.3	288.1	19.7	288.1	17.7
O 1s 3	531.1	34.5	531.1	41.1	531.2	38.2
N 1s 1	401.0	16.7	401.0	19.6	400.9	17.7
selected atomic ratios						
C/O_stoich._		1.5		1.0		
C/O_exp._		1.4		1.0		1.1
O/N_stoich._		2.0		2.0		
O/N_exp_		2.1		2.1		2.1
C/N_stoich._		3.0		2.0		
C/N_exp._		2.9		2.0		2.4

a Selected experimental (exp.) atomic ratios are presented and compared against those expected from stoichiometry (stoich.). Assignments and comments are included in the text.

b Set to 285.0 eV in the α-aminobutyric acid C 1s fitting (used as BE reference for charge accumulation correction). The charge correction of the remaining samples is described in the text.

Calibrations were performed with an α-aminobutyric acid (^+^NH_3_CH_2_CH_2_CH_3_COO^–^) nanolayer produced similarly to the remaining samples (see Section 2.5). The C 1s region, for the three pure amino acids—glycine, alanine, and α-aminobutyric acid—was fitted with as many peaks as the number of carbons in each molecule. For pure α-aminobutyric acid (not shown), four peaks were fitted under the constraints of all having equal areas, fwhm and Gaussian–Lorentzian products (GL%). The fitting was successful without the need of any additional peaks, attesting the absence of carbonaceous surface contamination. The lowest BE peak, assigned to the carbon in the −CH_3_ group (C 1s 1), was set to 285 eV. Consequently, the peak corresponding to the CH_3_-bonded −CH_2_– group (C 1s 2) was located 0.13 eV higher, at ∼285.1 eV. The nitrogen-bonded α-carbon peak (C 1s 3) was centered at 286.0 eV, while the highest BE peak (C 1s 4), at ∼288.1 eV, was assigned to the carboxylate (−COO^–^) group. This BE is consistent with complete ionization, as the carboxylic group (−COOH) would exhibit a BE higher than 289 eV.^79^ For this same sample, both O 1s and N 1s peaks were fitted with single symmetric peaks. The N 1s peak, centered at 401.0 ± 0.1 eV, is significantly higher than typical amines (centered at 399–400 eV)^79^ but slightly lower than the positively charged nitrogen (centered at ∼401.5 eV).^79^ This is compatible with a -NH_3_^+^ group involved in hydrogen bonding, acting as electron acceptor from an oxygen lone pair in the carboxylate group. The C 1s photoelectron data from the carboxylate group further confirms the zwitterionic form of the molecules, stabilized by hydrogen bonds. The O 1s peak, centered at 531.1 ± 0.1 eV, is characteristic of a carboxylate ion with the delocalized negative charge, where the two oxygen atoms are equivalent, unlike in the carboxylic group. For the remaining pure amino acids (alanine and glycine) and mixture samples, the highest C 1s BE was set to the same value as the one found for the α-aminobutyric acid, ∼288.1 eV, corresponding to the carbon in the carboxylate ion (C 1s 4). Using this reference, all samples exhibited a peak at 286.0 ± 0.2 eV, assigned to the N^+^-bonded α-carbon (C 1s 3) with the same area as the reference peak. In all samples except for pure glycine, an additional peak at 284.7 ± 0.2 eV was fitted (C 1s 2), corresponding to the alanine −CH_3_ carbon (Table 2).

The difference in binding energy between the C 1s3 (α-carbon) and C 1s4 (carboxylate carbon) atoms reflects the difference in electron density between the two; specifically, a higher BE indicates a lower electronic density at that atom. It allows to infer that the dipole moment of the C3–C4 bond is larger in glycine than in alanine, with the mixture exhibiting an intermediate value. Furthermore, in the alanine + glycine sample, the dipole moment of the C3–C4 bond becomes the same for the alanine and glycine molecules. This equalization is due to the convergence in the BE of the C3 (α-carbon), as the C 1s3 peak in the mixture sample can be fitted with a single peak, whose fwhm is indistinguishable, within the experimental error, from that of the individual samples (1.794 eV for alanine, 1.644 eV for glycine, and 1.714 eV for the alanine + glycine mixture). In other words, upon codeposition, the α-carbons of the amino acids become indistinguishable. In particular, the α-carbon of glycine has its electronic density decreased, which is reproduced in the transition state of the glycine decarboxylation reaction in an alanine-like environment, as we demonstrate computationally elsewhere.^68^

In the mixture sample, the ratio of the peak areas corresponding to the alanine −CH_3_ carbon (C 1s 2) and the COO^–^ carbon (C 1s 4) was used to estimate the surface composition of the alanine + glycine mixture. Let *A*2 and *A*4 denote the relative areas of the C 1s 2 and C 1s 4 peaks, respectively, and *G* and *A* the molar fractions of glycine and alanine, respectively, the following equations can then be established:

$$\frac{A2}{A4}=\frac{A}{G+A}=\frac{1}{G/A+1}=\frac{8.6}{17.7}$$ 1

G+A=1 2

Solving these two equations yields molar fractions of glycine at *G* = 0.51 and of alanine at *A* = 0.49, demonstrating an approximate 1:1 ratio in the alanine + glycine mixture. It is worth recalling that the depth analyzed by XPS is around 10 nm; therefore, this composition may not represent the entire film.

To further explore quantitative results, we referred to literature values where the density is reported as 1.42 g cm^–3^ for alanine and 1.61 g cm^–3^ for glycine.^90^ Given their molar masses are 89.09 and 75.07 g mol^–1^, respectively, this results in specific molarities of 0.01594 mol cm^–3^ for alanine and of 0.02144 mol cm^–3^ for glycine. Since each molecule contains only one nitrogen atom, and all spectrometer analysis conditions were kept constant across the samples, the area ratio *A*(N 1s of alanine)/*A*(N 1s of glycine) should equal the ratio of the specific molarities 0.01594/0.02144 = 0.743. The fitted peak areas were 7218.59 cps·eV for alanine and 9693.87 cps·eV for glycine, yielding a ratio of 0.744, confirming our expectation. The density of α-aminobutyric acid was not readily available in the literature (only an estimation of 1.23 g cm^–3^ is provided),^90^ making it challenging to compute its exact value. Following the same assumptions as above and using the N 1s peak area of 6102 cps·eV, its molarity is calculated as

$$\frac{6102}{9693.87}\times 0.02144=0.0135\;\mathrm{mol}\;{\mathrm{cm}}^{-3}$$

With a molar mass of α-aminobutyric acid of 103.12 g mol^–1^, we obtain a density for its nanolayer of 1.39 g cm^–3^. Applying the same procedure to the alanine + glycine sample yields a density of 1.60 g cm^–3^, slightly lower than that of pure glycine. Considering that the weighted average density for a 1:1 mixture of alanine and glycine would be 1.52 g cm^–3^, the measured density indicates proper mixing and a new arrangement of the amino acids upon codeposition. In this new arrangement, both amino acids attain the same α-carbon electron density.

### 3.4 Astrobiological Implications

The intramolecular electron density redistribution—due to the alanine-glycine interaction—may influence the photochemical behavior of the individual molecules. Previous studies have shown that the photodegradation pathway of amino acids starts with their decarboxylation, i.e., the cleavage of their carboxylate group from the α-carbon.^20,39,41,42,91^ The carboxylate fragment rearranges to form carbon dioxide, while the remaining molecule becomes a radical with its charge localized on the α-carbon. To justify the differences in degradation rates between alanine and glycine, ten Kate et al.^91^ argued that the kinetics of amino acid photodegradation are dictated by the stability of the α-carbon radical. Crucially, our XPS analysis revealed that the electron densities of the alanine and glycine α-carbons converge upon codeposition. And in another work, we demonstrated that the codeposition of alanine and glycine also converged their photodegradation rates compared to the individual irradiations of the amino acids at the same conditions^68^; a correlation which backs the proposal by ten Kate et al.^91^ Additionally, we observed a 10-fold increase in the degradation rate of glycine when codeposited with alanine. This substantial increase in the photodegradation further exemplifies how organic interactions can promote unexpected photochemistry of prebiotic molecules at planetary environments of interest.

In this work we considered the interaction between the two simplest α-amino acids in the most unbiased ratio (1:1), a simplified organic composition that prevented the properties of one molecular species to overshadow their interaction. Despite not being based on the known composition of any astrochemical or astrobiological scenario of interest, this study served to demonstrate that organic interactions can dictate the depositional arrangements, IR signatures, and electronic properties of biosignatures. As future work, we should evaluate the alanine-glycine interaction at different ratios and progressively include other organic molecules. We believe such progressive effort will further uncover how the detection and evolution of molecules of interest depend on their organic environment.

The gained knowledge may not only impact the characterization of the interstellar medium refractory materials and the in situ characterization of the Titan’s cryovolcanic regions described above, but they can also impact the properties and dynamics of organic evaporites produced from the concentration of desiccating lakes and seas and subsequent precipitation of their organic solutes. Characterizing these organic deposits where organic molecules are in close interaction may reveal the original composition of the solutions from which they precipitated. Therefore, they are of astrobiological interest to constrain the composition of Titan’s hydrocarbon lakes.^92–94^ For the same reason, the lake sediments of Titan’s north polar region have recently been proposed as a landing site and second-priority target for an ESA Large-class mission.^95^ Our results support this proposal by demonstrating the potential complexity of the organic evaporites and emphasizing that their in situ signatures may not be easily predicted.

## 4 Conclusions

Upon codeposition of alanine and glycine, we obtained different deposition behaviors, different IR signatures, and different electronic density distributions compared to what could be predicted from the study of the individual molecules. The characterization techniques unanimously suggested that both amino acids evolved toward the properties of each other. From IR spectroscopy, we inferred that the shared vibrational modes of alanine and glycine coupled in the alanine + glycine mixture sample. AFM studies showed that the alanine + glycine nanolayer attained a morphology intermediate between that of amorphous alanine and crystalline glycine, despite the glycine crystalline islands detected at its surface by nanoFTIR. XPS measurements showed how these interactions between organic molecules could alter their intramolecular electron density distributions and likely lead to unexpected photochemical behaviors.

It has been shown that interactions with inorganic surfaces lead to significant changes in the properties of molecules of interest. Herein, we have demonstrated that organic interactions can produce equally important effects. Further studies to better understand the organic interactions are thus necessary; either the interactions between different amino acids, or those between other prebiotic molecular families such as sugars and nucleobases. Also, the studied alanine-glycine system merits further enquiries. Would mixing ratios different than 1:1 equally influence the electronic densities of the amino acid α-carbons? We studied both alanine and glycine in their zwitterionic form. Would the interaction between neutral amino acids, expected at low-temperature depositions, produce equally extensive and impactful conformational variations? Also, complementary computational studies should constrain the extent and implications of the amino acid interactions. In another work, we show through ab initio quantum chemistry calculations how they change substantially the photodegradation rate of glycine.^68^ Future studies should seek to constrain the proper molecular interactions that produce the effects detailed above and to extrapolate them to other organic interactions.

Overall, if the interaction between compounds as simple and as similar as alanine and glycine leads to discernible differences in their morphological, spectroscopic, and electronic behaviors, what can we expect from the interactions between much more dissimilar molecules? To answer that, we suggest holistic approaches to future characterization and photochemistry studies of organic matter.

## Supplementary Material



## Notes

Further information on the transmission FT-IR features of all samples (including the evolution to low temperatures) and nanoFTIR analysis of the alanine + glycine sample surface crystals (PDF)

## Abbreviations

AFM: atomic force microscopy
nanoFTIR: nanoscale Fourier-transform infrared spectroscopy
XPS: X-ray photoelectron spectroscopy
CaF_2_: calcium fluoride
RMS: root-mean-square
BE: binding energy
