# Supplementary material for: Characterization of Amino Acid Nanolayers and Their Interactions under Simulated Planetary Conditions
Source: ACS Earth Space Chem. 2025 Feb 11;9(2):356–68. doi: 10.1021/acsearthspacechem.4c00334 (PMC12818834; doi:10.1021/acsearthspacechem.4c00334)
Supplement: Supplementary file 1 [file sp4c00334_si_001.pdf]

Characterization of amino acid nanolayers and their interactions under simulated planetary conditions

Diogo Gonçalves[1,2], Florence Hofmann[3], Janina Drauschke[3], Severin Wipf[3], Riccardo Giovanni Urso[4], Ana M. Ferraria[2,5], Ana M. Botelho do Rego[2,5], Jana Bocková[6], Cornelia Meinert[6], Andreas Elsaesser[3], Bruno Pedras[2,5†], Zita Martins[1*†]

[1]Centro de Química Estrutural, Institute of Molecular Sciences and Department of Chemical Engineering, Instituto Superior Técnico, Universidade de Lisboa, Av. Rovisco Pais 1, 1049-001 Lisbon, Portugal

[2]Institute for Bioengineering and Biosciences and Department of Chemical Engineering, Instituto Superior Técnico, Universidade de Lisboa, Av. Rovisco Pais 1, 1049-001 Lisbon, Portugal

[3]Freie Universität Berlin, Department of Physics, Experimental Biophysics and Space Science, Arnimallee 14, 14195, Berlin, Germany

[4]INAF-Osservatorio Astrofisico di Catania, Via Santa Sofia 78, 95123, Catania, Italy

[5]Associate Laboratory i4HB-Institute for Health and Bioeconomy, Instituto Superior Técnico, Universidade de Lisboa, Av. Rovisco Pais 1, 1049-001 Lisbon, Portugal

[6]Université Côte d'Azur-CNRS, ICN, UMR7272, 06108 Nice, France

*Corresponding author: zita.martins@tecnico.ulisboa.pt

†These authors share senior authorship

Table of contents

Further transmission FT-IR characterization ... 3

Amorphous and crystalline alanine thin layers ... 3

Spectral difference between pure-sample average and alanine+glycine spectrum ... 4

Interaction at low temperatures ... 5

NanoFTIR characterization of spatial features ... 8

References ... 11

Further transmission FT-IR characterization

Amorphous and crystalline alanine thin layers

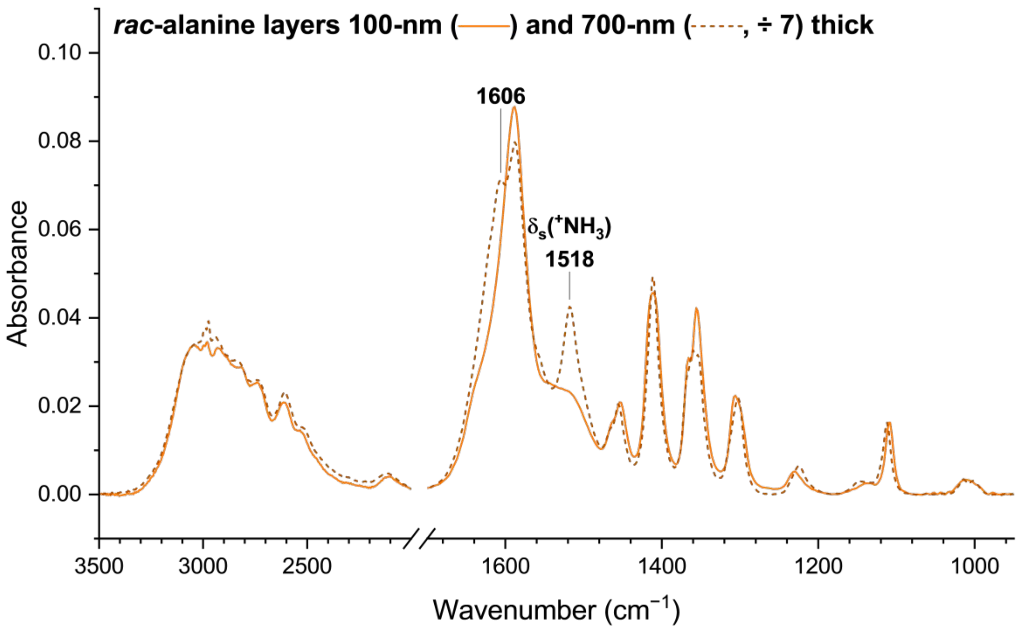

Figure S1. *Comparison between transmission FT-IR spectra of* rac-*alanine layers of 100-nm and 700-nm thickness deposited in our setup. The absorbance of the latter was divided by seven to facilitate the comparison of the band shapes in the two samples. Despite a near-perfect fit between the two layers, sharper bands at 1606 and 1518 cm* $^{-1}$ *suggest a higher crystallinity in the 700-nm layer sample.*

Spectral difference between pure-sample average and alanine+glycine spectrum

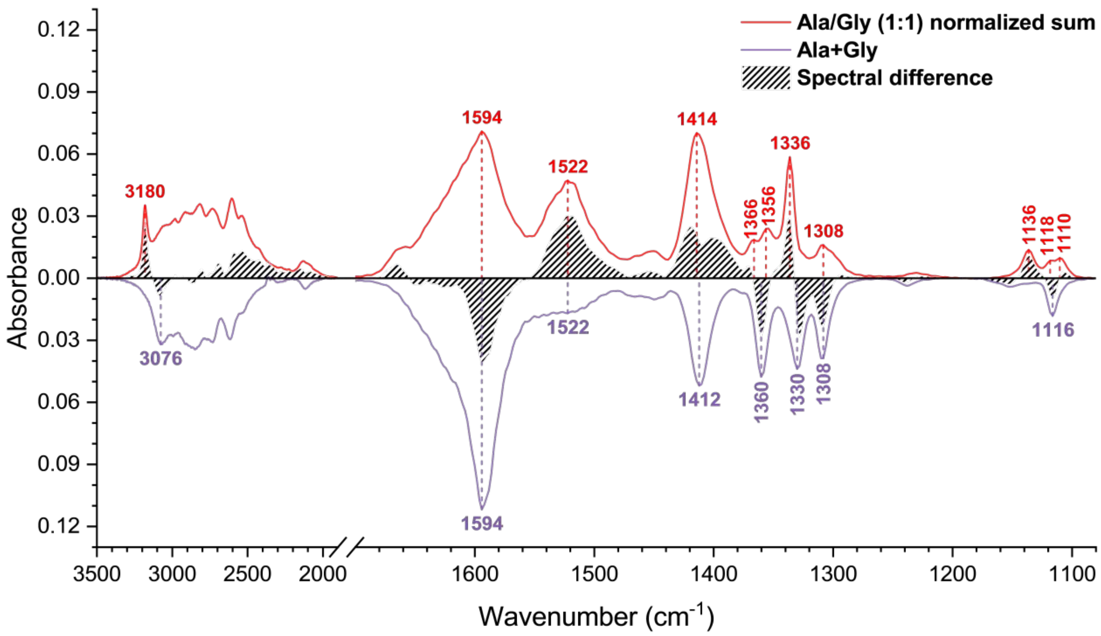

Figure S2. *Comparison between the normalized sum (1:1) of the pure alanine and pure glycine transmission FT-IR spectra (top) and the alanine+glycine mixture sample spectrum (bottom). The difference between the two spectra is depicted in stripe pattern.*

We computed the arithmetic difference (**Figure S2**, stripe pattern) between the spectrum obtained from the normalized sum of the pure alanine and glycine spectra—i.e., their average at the 1:1 ratio, **Figure S2**, top—and the alanine+glycine mixture sample spectrum (**Figure S2**, bottom). The opposite signs of the spectral differences at 3180 cm^{-1} and at 3076 cm^{-1} reveal the evolution in the glycine $\nu_{as}(^{+}NH_3)$ vibrational mode induced by the new environment felt by the glycine molecules. That the alanine+glycine sample is, overall, less crystalline than the average of the two pure samples is signaled in the significant band intensity difference detected at 1522 cm^{-1}. On the other hand, the significant differences in band intensity at 1360 cm^{-1} and 1308 cm^{-1} (assigned to the -CH_3 and -CH- alanine groups, respectively) signal the increase in crystallinity in the arrangement of the alanine molecules. Lastly, the significant differences at 1136–1110 cm^{-1} arise from the coalescence of the $\rho(^{+}NH_3)$ vibrational modes of the single amino acids into a shared, single peak at 1116 cm^{-1}, demonstrating the vibrational coupling of the -$^{+}NH_3$ groups of both molecules. These spectral differences are consistent with the evolutions discussed in the main text.

Interaction at low temperatures

At decreasing temperatures, the spectroscopic features of the pure alanine and pure glycine samples evolved as previously reported,[1–5] showing an increase in energy in most vibrational modes (**Table S1**), with their bands becoming sharper and more intense. These band transformations likely influence their integrated absorptivity as well.[4,5] The higher vibronic resolution at low temperatures resolved the glycine broad bands encoding for the $\nu_s(COO^-)$ and $\delta_s(^+NH_3)$ vibrational modes into doublets. This could not be detected in the alanine sample, likely due to its amorphous arrangement. The alanine energy shifts during cooldown were uniform across all its modes, with the $\nu_{as}(^+NH_3)$ mode remaining unchanged—only increasing in intensity and sharpness. On the other hand, the crystalline glycine sample had the highest energy variations in the $-^+NH_3$ group, as previously observed,[3] justified by the contraction of the unit cell and shortening of the hydrogen bonds lengths.[5]

Table S1. *Shifts in band energy after cooldown of alanine and glycine samples from room temperature (RT) to 90 K.*

	Alanine		Glycine	
Assignment	RT $\bar{\nu}$ (cm^{-1})	$\Delta\bar{\nu}_{RT\rightarrow 90\,K}$ (cm^{-1})	RT $\bar{\nu}$ (cm^{-1})	$\Delta\bar{\nu}_{RT\rightarrow 90\,K}$ (cm^{-1})
$\nu(CC)$	-		894	+3*
$\rho(CH_2)$	-		916	0
$\rho(^+NH_3)$	1012	+4	-	
$\nu_{as}(CCN)$	-		1040	+3
$\rho(^+NH_3)$	1110	+3	1118, 1136	+2, +4
$\delta(CH)$	1134	+8	-	
$\rho(CH)$	1230	+5	-	
$\delta(CH)$	1306	+4	-	
$\omega(CH_2)$	-		1336	+1
$\delta_s(CH_3)$	1356, 1366	+2*	-	
$\nu_s(COO^-)$	1412	+2*	1414	-2, +4†
$\delta_s(^+NH_3)$	1522	-*	1522	+1, +5†,*
$\nu_{as}(COO^-)$	1588	0	1602	0*
$\delta_{as}(^+NH_3)$ + $\tau(^+NH_3)$	2108	+5*	2134	+30
$\nu_{as}(^+NH_3)$	3044	0	3180	+8

*Measurement with significant uncertainty due to low signal-to-noise ratio or overlap with water vapor absorption bands.
†Band splitting.

Upon cooling, the alanine+glycine sample evolved similarly to the individual amino acids. All absorption bands became sharper, more intense, and shifted to higher wavenumbers as the cooling proceeded (**Figure S3**). All shifts in peak position measured in the mixture sample were similar to their equivalent in the single amino acids (**Table**

S2). The peak shifts remained uniform across the absorption bands, suggesting a rigid conformation kept during cooling, particularly in the molecule-specific δ(CH), ω(CH_2) and δ_s(CH_3) modes (**Figure S3**). The vibrational modes whose evolution upon cooling differed in the alanine and glycine samples, most notably the δ_{as}($^+NH_3$) + τ($^+NH_3$) mode, exhibited an intermediate behavior in the mixture. The ν_{as}($^+NH_3$) mode, reflecting the average hydrogen bond strength, increased in intensity without any shift in the peak position, mirroring the behavior of the alanine sample. In contrast, this mode blue-shifted in the glycine sample. This observation supports the previous conclusion that alanine dictates the strength of the average hydrogen bonds in the mixture sample.

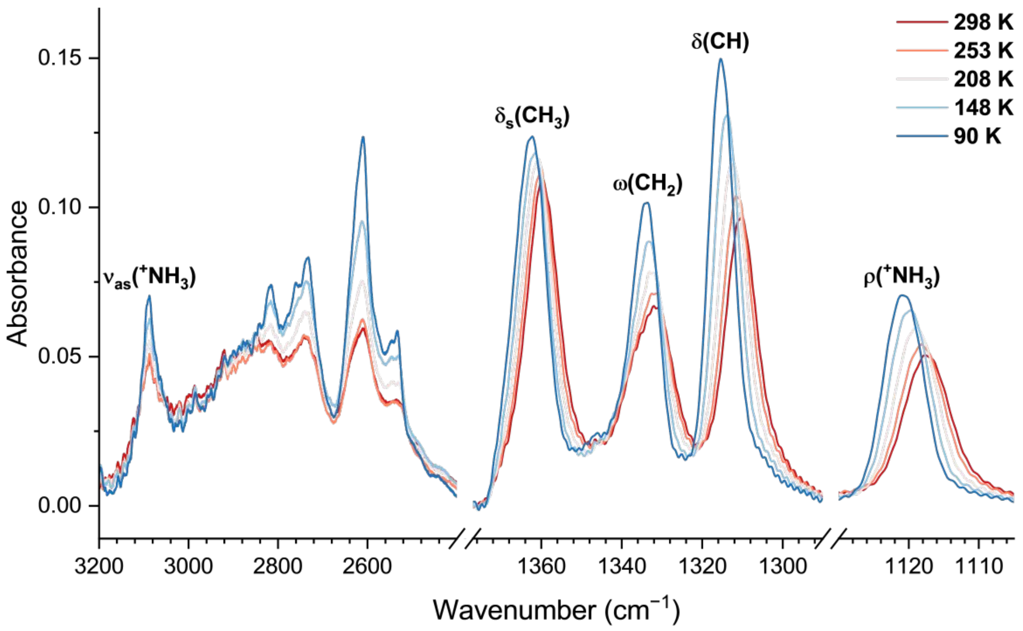

Figure S3. *Evolution of absorption features in the alanine+glycine sample upon cooling from room temperature to 90 K. A steady increase in intensity, energy and sharpness is seen across all modes, except for the N-H and C-H stretching vibrations whose energies remain constant.*

Crucially, the bands which best illustrate the combined vibrational modes of alanine and glycine, particularly the ρ($^+NH_3$) and ν_{as}($^+NH_3$) modes, remained sharp singlet bands upon cooling and did not resolve into separate contributions from the individual amino acids (**Figure S3**). Similarly, the 2530–3000 cm^{-1} region, while becoming better defined, did not split into separate alanine and glycine features. This confirms that the band combinations seen at room temperature were not thermal broadening artifacts; rather, they reflect genuine vibrational coupling between the amino acids.

Table S2. *Shifts in band energy upon cooling of the alanine+glycine sample from room temperature (RT) to 90 K and comparison with the evolution of the same vibrational modes in both single-amino acid (aa) samples.*

	**Alanine+Glycine**		Corresponding evolution in single-aa samples ($\Delta\bar{\nu}_{RT\rightarrow 90\ K}$)	
Assignment	RT (cm^{-1})	$\Delta\bar{\nu}_{RT\rightarrow(90\ K)}$ (cm^{-1})	**Alanine** (cm^{-1})	**Glycine** (cm^{-1})
$\rho(^{+}NH_3)$	1014	+5	+4	-
$\nu_{as}(CCN)$	1042	+5	-	+3
$\rho(^{+}NH_3)$	1116	+3	+3	+2, +4
Combination	1152	+8	-	-
$\rho(CH)$	1238	+5	+5, +12†	-
$\delta(CH)$	1308	+5	+4	-
$\omega(CH_2)$	1330	+3	-	+1
$\delta_s(CH_3)$	1360	+3	+2	-
$\nu_s(COO^{-})$	1412	+3	+2	-2, +4†
$\nu_{as}(COO^{-})$	1594	0	0	0
$\delta_{as}(^{+}NH_3) + \tau(^{+}NH_3)$	2116	+10	+5	+30
$\nu_{as}(^{+}NH_3)$	3076	0	0	+8

†Band splitting.

NanoFTIR characterization of spatial features

Transmission FT-IR and topographic analyses demonstrated that the alanine and glycine molecules in the mixture sample arranged differently than when deposited individually. To understand the extent of the interaction between the two amino acids, nanoscale Fourier-transform infrared spectroscopy (nanoFTIR) was used to investigate the spatial heterogeneities in the alanine+glycine nanolayer. This technique allows for a spatial resolution limited by the size of the tip apex, typically 20 nm,[6,7] sufficient to distinguish the alanine+glycine surface crystals from their background.

First, the morphology of the pure alanine and pure glycine nanolayers was homogeneous throughout the respective surfaces. The nanoFTIR absorption spectra of the alanine layer, measured at six different locations, agree exceptionally well with its far-field transmission FT-IR spectra (**Figure S4a**). In the 1700–1200 cm^{-1} spectral range, all major bands appear at the same wavenumbers as in the far-field absorption spectra, matching the band shapes and area ratios. Still, a significant difference lies in the doublet centered at 1608 cm^{-1}, likely arising from the enhanced sensitivity of nanoFTIR to molecular vibrations that oscillate perpendicular to the sample surface.[6] This interpretation is supported by the fact that the double-peaked band is reproduced in the reflection-absorption spectrum (**Figure S4a**). The latter is composed by the signal transmitted through the amino acid nanolayer and, crucially, the signal reflected from the nanolayers top surface. Like nanoFTIR, the reflected signal has higher sensitivity to vibrational modes perpendicular (*s*-polarized) to the plane of incidence[8–10] (which produces notable spectral differences to the transmitted signal[11]), suggesting that the doublet at 1608 cm^{-1} in the nanoFTIR spectra does arise from vibrational modes perpendicular to the nanolayer surface. The 1522 cm^{-1} band, which signals the crystallinity of the layer (**Figure S1**), appears weak and broad in all alanine nanoFTIR spectra. In most cases, it is barely distinguishable, suggesting that the predominantly amorphous nature is consistent across the alanine sample, irrespective of whether the locations are lower (L1, L2, and L4) or higher (L3, L5, and L6) within the nanolayer. This uniform amorphous structure, illustrated in **Figure S4a**, aligns with the significant variations in the total band area and in the doublet at 1608 cm^{-1} detected at different locations. These variations likely originate from differences in the number of molecules probed and in the average molecular orientation under the tip.

The glycine nanoFTIR spectra, on the other hand, presented peak intensities much more uniform across the probed locations, as expected for a crystalline sample. The nanoFTIR spectra closely matched the far-field transmission spectra, with some peak positions and band-area ratios slightly better predicted by the reflection-absorption spectrum (**Figure S4b**), for the reasons given above. Overall, the nanoFTIR hyperspectral imaging of both alanine and glycine samples provided the confidence needed to proceed with the characterization of the spatial features in the alanine+glycine mixture, drawing analogies to the far-field absorption spectra.

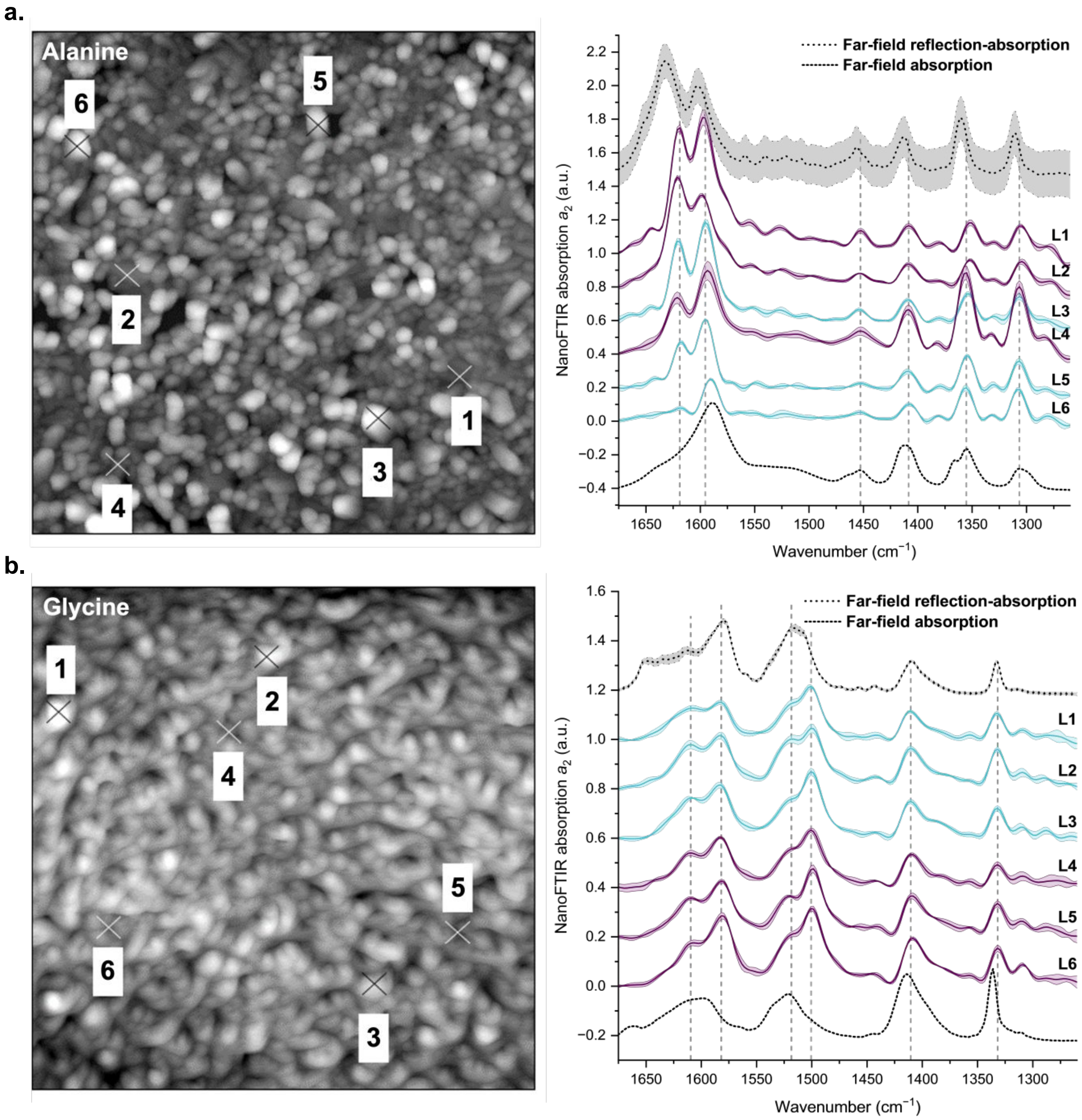

Figure S4. *NanoFTIR absorption spectra of six different areas of the alanine (**a**) and glycine (**b**) samples. The spectra are overlaid with the far-field reflection-absorption (dotted) and absorption (dashed) spectra, showing close agreement (grey dashed lines). The nanoFTIR absorption spectra showcase the variability expected in the amorphous organization of the alanine nanolayer, and the uniformity expected from the crystalline structure of the glycine sample.*

The alanine+glycine crystals were uniformly distributed throughout the surface of the mixture nanolayer. As the nanoFTIR spectra from these crystals closely matched the far-field absorption spectrum of pure glycine, rather than that of alanine+glycine (**Figure S5**, locations L1–3), we have understood that they were largely enriched in glycine. This was evident from the relative prominence, in the crystalline areas, of the ω(CH_2) mode at 1332 cm^{-1}, the asymmetric band shape at 1408 cm^{-1}, the sharp δ_s($^+NH_3$) band at 1522 cm^{-1}, and the δ_{as}($^+NH_3$) band at 1660 cm^{-1}, all characteristic of glycine. The background (locations L4–6) is neither enriched in alanine nor glycine but is instead composed of both amino acids at a similar ratio to that measured in the far-field spectra (**Figure S5**, locations L4–6). This is clearest in the peak-height ratios between the alanine-specific δ_s(CH_3) and δ(CH) modes at 1355 and 1307 cm^{-1}, respectively, and the glycine-specific mode at 1327 cm^{-1}. These peak-height ratios closely

match across the background locations and the alanine+glycine far-field transmission spectra. This suggests that the glycine crystal islands represent only a minute fraction of the total nanolayer, with most of the glycine interacting with alanine in homogenous domains. However, some glycine-induced crystallinity, as described in section 3.1.2, was observed at location L4. Although the ratio of amino acid-specific bands in the 1360–1300 cm^{-1} range resembles that in L5 and L6, a sharp band at 1496 cm^{-1} hints at an alanine-glycine interaction in a more crystalline phase.

In summary, nanoFTIR analysis demonstrated that while glycine tends to segregate into dispersed crystalline islands during deposition, most of it does interact with alanine within the bulk composition of the alanine+glycine nanolayer.

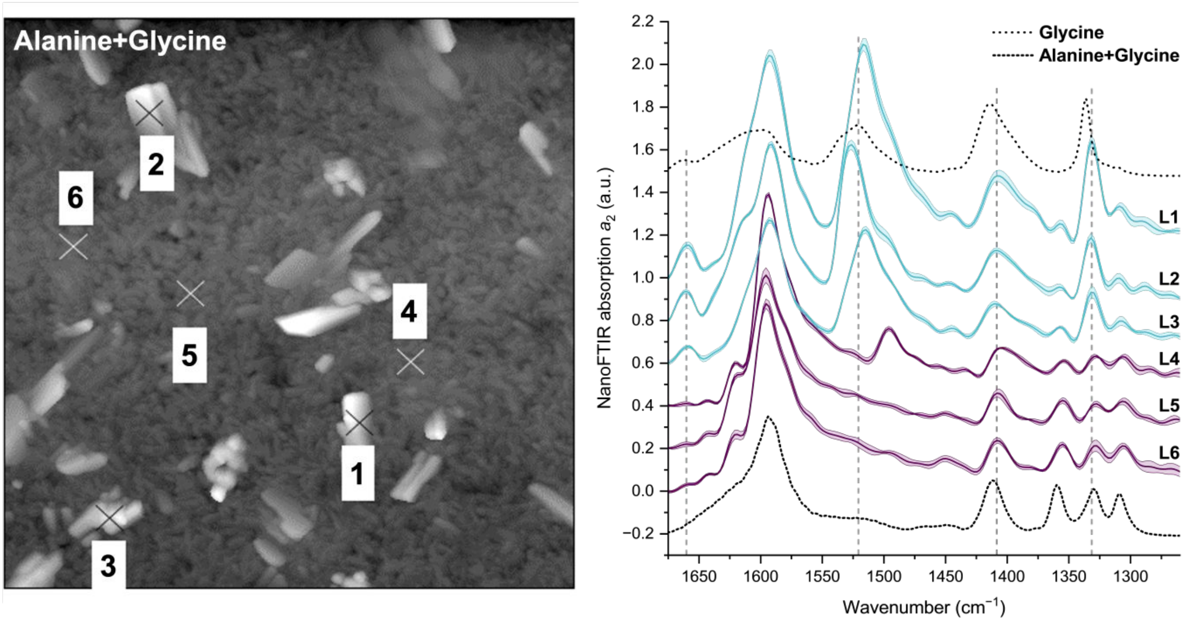

Figure S5. *NanoFTIR absorption spectra of six different areas of the alanine+glycine sample. The spectra at three crystalline locations (L1, L2, and L3) are similar to the far-field absorption spectrum of glycine (dotted). The clearest spectral similarities are observed in the* $\delta_{as}(^{+}NH_3)$ *(1660 cm*$^{-1}$*),* $\delta_s(^{+}NH_3)$ *(1522 cm*$^{-1}$*),* $\nu_s(COO^-)$ *(1408 cm*$^{-1}$*) and* $\omega(CH_2)$ *(1332 cm*$^{-1}$*) modes, as indicated by the grey dashed lines. The nanoFTIR absorption spectra of the "background" locations (L4, L5, and L6) closely resemble the far-field spectrum of the alanine+glycine sample (dashed). Although the crystals are enriched in glycine, glycine is also spread throughout the bulk composition of the nanolayer.*

References

(1) Rozenberg, M.; Shoham, G.; Reva, I.; Fausto, R. Low-Temperature Fourier Transform Infrared Spectra and Hydrogen Bonding in Polycrystalline l-Alanine. *Spectrochim Acta A Mol Biomol Spectrosc* **2003**, *59* (14), 3253–3266. https://doi.org/10.1016/S1386-1425(03)00159-8.

(2) Minkov, V. S.; Chesalov, Y. A.; Boldyreva, E. V. A Study of the Temperature Effect on the IR Spectra of Crystalline Amino Acids, Dipeptids, and Polyamino Acids. VI. L-Alanine and DL-Alanine. *Journal of Structural Chemistry* **2010**, *51* (6), 1052–1063. https://doi.org/10.1007/S10947-010-0162-4.

(3) Chernobai, G. B.; Chesalov, Y. A.; Burgina, E. B.; Drebushchak, T. N.; Boldyreva, E. V. Temperature Effects on the IR Spectra of Crystalline Amino Acids, Dipeptides, and Polyamino Acids. I. Glycine. *Journal of Structural Chemistry* **2007**, *48* (2), 332–339. https://doi.org/10.1007/S10947-007-0050-8.

(4) Iglesias-Groth, S.; Cataldo, F. Integrated Molar Absorptivity of Mid- and Far-Infrared Spectra of Glycine and Other Selected Amino Acids. *Astrobiology* **2021**, *21* (5), 526–540. https://doi.org/10.1089/AST.2020.2307.

(5) Iglesias-Groth, S.; Cataldo, F. Integrated Molar Absorptivity of Mid- and Far-Infrared Spectra of Alanine and a Selection of Other Five Amino Acids of Astrobiological Relevance. *Astrobiology* **2022**, *22* (4), 462–480. https://doi.org/10.1089/AST.2021.0094.

(6) Amenabar, I.; Poly, S.; Nuansing, W.; Hubrich, E. H.; Govyadinov, A. A.; Huth, F.; Krutokhvostov, R.; Zhang, L.; Knez, M.; Heberle, J.; Bittner, A. M.; Hillenbrand, R. Structural Analysis and Mapping of Individual Protein Complexes by Infrared Nanospectroscopy. *Nature Communications 2013 4:1* **2013**, *4* (1), 1–9. https://doi.org/10.1038/ncomms3890.

(7) Huth, F.; Govyadinov, A.; Amarie, S.; Nuansing, W.; Keilmann, F.; Hillenbrand, R. Nano-FTIR Absorption Spectroscopy of Molecular Fingerprints at 20 Nm Spatial Resolution. *Nano Lett* **2012**, *12* (8), 3973–3978. https://doi.org/10.1021/NL301159V.

(8) Urso, R. G.; Scirè, C.; Baratta, G. A.; Compagnini, G.; Palumbo, M. E. Combined Infrared and Raman Study of Solid CO. *Astron Astrophys* **2016**, *594*, A80. https://doi.org/10.1051/0004-6361/201629030.

(9) Yang, C.; Wöll, C. Infrared Reflection-Absorption Spectroscopy (IRRAS) Applied to Oxides: Ceria as a Case Study. *Surf Sci* **2024**, *749*, 122550. https://doi.org/10.1016/J.SUSC.2024.122550.

(10) Greenler, R. G.; Snider, D. R.; Witt, D.; Sorbello, R. S. The Metal-Surface Selection Rule for Infrared Spectra of Molecules Adsorbed on Small Metal Particles. *Surf Sci* **1982**, *118* (3), 415–428. https://doi.org/10.1016/0039-6028(82)90197-2.

(11) Chabal, Y. J. Surface Infrared Spectroscopy. *Surf Sci Rep* **1988**, *8* (5–7), 211–357. https://doi.org/10.1016/0167-5729(88)90011-8.
